# Association between polymorphisms of *TAS2R16* and susceptibility to colorectal cancer

**DOI:** 10.1186/s12876-017-0659-9

**Published:** 2017-09-15

**Authors:** Jonathan Barontini, Marco Antinucci, Sergio Tofanelli, Maurizio Cammalleri, Massimo Dal Monte, Federica Gemignani, Pavel Vodicka, Roberto Marangoni, Ludmila Vodickova, Juozas Kupcinskas, Veronika Vymetalkova, Asta Forsti, Federico Canzian, Angelika Stein, Victor Moreno, Nicola Mastrodonato, Francesca Tavano, Anna Panza, Roberto Barale, Stefano Landi, Daniele Campa

**Affiliations:** 1 Department of Biology, University of Pisa, Via Derna 1, 56100 Pisa, Italy; 2 Department of Molecular Biology of Cancer, Institute of Experimental Medicine, Academy of Science of Czech Republic, Prague, Czech Republic; 3 Institute of Biology and Medical Genetics, 1st Medical Faculty, Charles University, Prague, Czech Republic; 4 Biomedical Centre, Medical School Pilsen, Charles University in Prague, Pilsen, Czech Republic; 5 Department of Gastroenterology, Lithuanian University of Health Sciences, Kaunas, Lithuania; 6 Genomic Epidemiology Group, German Cancer Research Center (DKFZ), Heidelberg, Germany; 7 Division of Molecular Genetic Epidemiology, German Cancer Research Center (DKFZ), Heidelberg, Germany; 8 Cancer Prevention and Control Program, Catalan Institute of Oncology (ICO), IDIBELL, CIBERESP and Department of Clinical Sciences, Faculty of Medicine, University of Barcelona, Barcelona, Spain; 9 Division of Gastroenterology and Research Laboratory, IRCCS Scientific Institute and Regional General Hospital “Casa Sollievo della Sofferenza”, San Giovanni Rotondo, Italy

**Keywords:** Taste receptors, *TAS2R16*, Polymorphisms, Colon cancer, Rectal cancer, Colorectal cancer, Cancer risk, Genetic association study

## Abstract

**Background:**

Genetics plays an important role in the susceptibility to sporadic colorectal cancer (CRC). In the last 10 years genome-wide association studies (GWAS) have identified over 40 independent low penetrance polymorphic variants. However, these loci only explain around 1‑4% of CRC heritability, highlighting the dire need of identifying novel risk loci. In this study, we focused our attention on the genetic variability of the *TAS2R16* gene, encoding for one of the bitter taste receptors that selectively binds to salicin, a natural antipyretic that resembles aspirin. Given the importance of inflammation in CRC, we tested whether polymorphic variants in this gene could affect the risk of developing this neoplasia hypothesizing a role of *TAS2R16* in modulating chronic inflammation within the gut.

**Methods:**

We performed an association study using 6 tagging SNPs, (rs860170, rs978739, rs1357949, rs1525489, rs6466849, rs10268496) that cover all *TAS2R16* genetic variability. The study was carried out on 1902 CRC cases and 1532 control individuals from four European countries.

**Results:**

We did not find any statistically significant association between risk of developing CRC and selected SNPs. However, after stratification by histology (colon vs. rectum) we found that rs1525489 was associated with increased risk of rectal cancer with a (P_trend_ of = 0.0071).

**Conclusions:**

Our data suggest that polymorphisms within *TAS2R16* gene do not have a strong influence on colon cancer susceptibility, but a possible role in rectal cancer should be further evaluated in larger cohorts.

**Electronic supplementary material:**

The online version of this article (10.1186/s12876-017-0659-9) contains supplementary material, which is available to authorized users.

## Background

Colorectal cancer (CRC) is the third most commonly diagnosed cancer worldwide [1]. Several environmental/life style risk factors have been identified for CRC, such as obesity, sedentary behavior, high red meat consumption, high intake of hyper caloric foods, diets rich in fat and poor in fiber, tobacco smoking, alcohol consumption and diabetes [2]. In addition gut inflammation, due to chronic conditions such as ulcerative colitis and Crohn’s disease, is an established risk factor [2]. Also genetics is hypothesized to play an important role for the sporadic form. In the last 10 years genome-wide association studies (GWAS) have identified over 40 independent low penetrance polymorphic variants [3]. Besides GWAS, an overwhelming number of hypothesis-driven studies carried out on specific candidate genes highlighted variants belonging to multiple pathways to be connected to CRC risk. The majority of the studies focused on SNPs within DNA repair genes [4], xenobiotic metabolism and transport genes [5–14], inflammatory response genes [15–17], hormone metabolisms [18, 19] and more recently miRNAs [20, 21]. Our group and others have also analyzed the possible involvement of taste receptor genes in CRC risk [22–24]. In the human genome, there are 25 functional genes and 11 pseudogenes belonging to the *TAS2Rs* gene family. It has already been ascertained that polymorphic variants in these genes are responsible for the variability observed in several human phenotypes and life habits such as alcohol consumption [25, 26], and nicotine dependence [27]. However, taste receptors seem to have also less obvious functions. In fact taste receptors certainly do not only have a role in taste perception, as documented by their ectopic expression in a variety of extra-oral tissues including gut [28–31]. In this study we focused our attention on the genetic variability of the *TAS2R16* gene whose encoded receptor selectively binds to salicin [32], a natural anti-inflammatory and analgesic agent extracted from willow bark and resembling aspirin [33]. Given the similarities between salicin and aspirin it is reasonable to postulate that variants within the *TAS2R16* gene could affect the receptor activity following its binding with ligands such as salicin or aspirin. In particular, aspirin intake is an established factor reducing the risk to develop CRC. In fact, there are evidences from case-control and cohort studies indicating benefit from long term use of aspirin at low dose in colorectal cancer chemoprevention [34]. This effect could be elicited through an anti-inflammatory activity or to the binding to specific receptors in the gut. Furthermore, there are indications that regular use of aspirin is associated with improved survival for patients with colorectal cancer [35]. The hypothesis is corroborated by a previous study (Campbell and colleagues) conducted on two different African populations, describing that the polymorphic variant rs846664 within TAS2R16 confers different sensitivities to salicin [36]. Unfortunately, this finding could not be replicated among Caucasians since this SNP is monomorphic in this population. However, according to this base of knowledge, it could be hypothesized that *TAS2R16* could modulate chronic inflammation in the gut. Alternatively, the receptor, upon binding with its natural ligands, could affect the individual risk of cancer with mechanisms similar to those observed following a long term aspirin intake. Thus, in the present work we tested whether polymorphic variants within *TAS2R16* could affect the risk of developing CRC. The study was carried out in a large cohort of European individuals.

## Methods

### Study population

A total of 2252 CRC case subjects and 1630 controls were collected in four European countries (Czech Republic, Italy, Lithuania and Spain). CRC patients had histological verification of colorectal adenocarcinoma. Controls were collected in the same hospitals and in the same time frame as the cases among hospitalized individuals without neoplastic diseases. A more detailed description of the sampling and collection methods has been provided elsewhere [9, 37–39]. For the cases we collected information about gender, age at diagnosis, tumor localization (colon or rectum), while for the controls we have information on gender and age at sampling.

### SNPs selection

We used tagging SNPs to cover all the common genetic variability of *TAS2R16*. We selected the gene region and added 4 k base pairs on the 5 prime and 3 prime directions. According to HapMap 3 database, within the selected region there are 24 known common SNPs with a MAF > 0.05 among Caucasians. Using the software Haploview [40] we selected six tagging SNPs by setting the LD threshold at r^2^ ≥ 0.8. The final selection of the SNPs resulted in rs860170, rs978739, rs1357949, rs1525489, rs6466849, and rs10268496 covering 87% of the common genetic variability.

### Sample preparation and genotyping

DNA was extracted from whole blood. Samples were kept frozen until used. Genotyping was performed on 384 well plates using the KASP (LGC, Teddington, UK) and TaqMan (Thermo Fisher, Waltham, MA USA) genotyping chemistry. In each plate there was DNA from cases and controls in order to avoid bias due to genotyping. We included a mean of 15 duplicated samples for plate to be used as quality controls. After the PCR the plates were “read” via the spectrophotometer Fluostar Omega (Ω) (BMG.LABTECH®) and genotyping calls were done using the KlusterCaller software (LGC Group Teddington, UK).

### Statistical analysis

The observed genotype frequencies of all SNPs were tested for deviation from Hardy–Weinberg equilibrium (HWE) in controls. The association between the genotypes of all polymorphisms and CRC risk was estimated using an unconditional logistic regression computing odds ratios (OR), 95% confidence intervals (95% CIs) and *P*-values. The common allele among the controls was assigned as the reference category and the co-dominant inheritance model was assessed. All analyses were adjusted for age (at diagnosis for cases and at sampling for controls), gender and country origin. We also performed stratified analyses for country of origin and tumor site. The significance level in each test was adjusted according to the Bonferroni’s correction, namely adjusted P = α/number of individual tests (0.05/6 = 0.008).

### Bioinformatic tools

We carried out bioinformatic analyses to assess the possible functional effect of the studied polymorphisms, for example to detect if the nucleotide sequences of the polymorphisms are targets of transcription factors or if they are situated in DNase sensitivity region. Specifically we have used RegulomeDB V1.1 [41] and Haploreg V4.1 [42]; to test whether the polymorphisms could have a known regulatory impact. Additionally, we used the data available in the GTEx project V6 to identify expression of quantitative trait loci (eQTLs) [43].

## Results

### Data filtering and quality control

We examined 6 SNPs belonging to the *TAS2R16* gene to assess their possible role in the risk of developing CRC. We have genotyped up to 3882 individuals (2252 CRC cases and 1630 controls) enrolled in the Czech Republic, Lithuania, Italy and Spain. The distributions of the genotype frequencies for all the variants included in the study were in accordance with HWE. Four hundred forty-eight (448) subjects (346 CRC cases 102 controls) for which we did not have covariates data or that did not pass quality control (call rate < 75%) were removed from subsequent analyses, leaving a total of 3434 subjects (1902 CRC cases and 1532 controls) for statistical analysis. All the relevant characteristics of the population used in the analyses are shown in Table 1. The average SNP call rate was 96.24% with a minimum of 98.16% (rs1525489) and a maximum of 99.66% (rs10268496). The analysis of the samples duplicated for quality control showed a concordance greater than 99%. All the frequencies, call rate and HWE values of each polymorphism are reported in Additional file 1: Table S1.

**Table 1:** Population in study

Country of origin	Colorectal Cancer Cases				Controls			
	Males	Females	Total	Mean Age	Males	Females	Total	Mean Age
Czech Republic	588	400	988	61.92	393	296	689	54.87
Lithuania	102	76	178	67.04	91	92	183	57.29
Italy	179	142	321	65.97	252	94	346	51.94
Spain	248	167	415	66.35	166	148	314	65.34
**Total**	**1117**	**785**	**1902**	**64.09**	**902**	**630**	**1532**	**56.69**

### Effect of SNPs on colorectal cancer risk

Overall, we did not observe any statistically significant association between selected SNPs and risk of CRC. The polymorphism with the lowest *p*-value was rs1525489 showing a p-trend of *P* = 0.084. Following the stratification by tumor site (colon / rectum) we found that rs1525489 showed a marginal/borderline significance with increased risk of rectal cancer at nominal level of (P_trend_ = 0.0071), while the associations between the SNPs and colon cancer were similar to those considering the two strata together (Table 2). However, performing a case-case analysis (colon vs. rectum) we did not observe any heterogeneity. When stratifying for country of origin we obtained one marginally significant value. In the Lithuanian case control study subjects the minor (C) allele of rs1525489 was associated with an increased risk of developing CRC with a nominal P_trend_ = 0.047. We observed the same trend also in the Spanish sub-population (*P* = 0.051). The stratified analyses are reported in Additional file 2: Table S2, Additional file 3: Table S3, Additional file 4: Table S4, and Additional file 5: Table S5.

**Table 2:** Association between colorectal cancer risk and SNPs in the *TAS2R16* region stratified by histology

SNP	Alleles (Major/minor)	Site	Case/Control^a^			MM vs Mm^b^	*P value*	MM vs mm^b^	*P value*	MM vs Mm+mm^b^	*P value*	MM + Mm vs mm^b^	*P* value	P trend
			MM	Mm	mm									
rs860170	A/G	All	785/653	846/681	245/184	1.04(0.9–1.22)	0.57	1.05(0.83–1.32)	0.69	1.05(0.9–1.21)	0.55	1.03(0.83–1.27)	0.82	0.37
		Colon	447/653	487/681	141/184	1.03(0.87–1.23)	0.73	1.07(0.82–1.4)	0.52	1.04(0.88–1.23)	0.64	1.06(0.82–1.35)	0.67	0.37
		Rectum	231/653	256/681	68/184	1.06(0.85–1.32)	0.61	1.02(0.74–1.43)	0.89	1.05(0.85–1.29)	0.64	0.99(0.73–1.36)	0.97	0.65
rs978739	A/G	All	830/693	777/678	182/156	0.97(0.83–1.13)	0.67	0.98(0.77–1.26)	0.89	0.97(0.84–1.12)	0.68	1(0.79–1.27)	0.99	0.65
		Colon	487/693	422/678	97/156	0.9(0.75–1.07)	0.22	0.88(0.65–1.17)	0.37	0.89(0.75–1.05)	0.18	0.92(0.7–1.22)	0.58	0.18
		Rectum	240/693	222/678	71/156	0.97(0.78–1.22)	0.82	1.36(0.97–1.9)	0.08	1.04(0.85–1.29)	0.68	1.37(1–1.89)	0.05	0.30
rs1357949	T/C	All	927/719	761/659	180/145	0.91(0.78–1.05)	0.20	0.95(0.74–1.23)	0.71	0.91(0.79–1.06)	0.22	1(0.78–1.27)	0.99	0.31
		Colon	512/719	443/659	110/145	0.97(0.81–1.15)	0.72	1.09(0.82–1.46)	0.54	0.99(0.84–1.17)	0.92	1.11(0.84–1.46)	0.46	0.98
		Rectum	292/719	211/659	50/145	0.78(0.63–0.97)	0.03	0.82(0.57–1.19)	0.30	0.79(0.64–0.97)	0.02	0.92(0.65–1.32)	0.66	0.06
rs1525489	T/C	All	1728/956	145/62	1/0	1.24(0.88–1.73)	0.22	-	-	1.22(0.88–1.70)	0.23	-	-	0.08
		Colon	992/956	77/62	0/0	1.22(0.83–1.8)	0.30	-	-	1.22(0.83–1.8)	0.30	-	-	0.31
		Rectum	505/956	53/62	1/0	1.59(1.03–2.43)	0.03	-	-	1.62(1.06–2.47)	0.03	-	-	**0.007**
rs6466849	G/A	All	1276/1024	524/452	62/52	0.97(0.83–1.14)	0.75	1.01(0.68–1.51)	0.95	0.98(0.84–1.14)	0.78	1.02(0.69–1.51)	0.92	0.40
		Colon	748/1024	284/452	36/52	0.88(0.73–1.05)	0.16	0.97(0.61–1.53)	0.88	0.88(0.74–1.06)	0.18	1(0.64–1.58)	0.99	0.16
		Rectum	376/1024	156/452	22/52	1(0.8–1.26)	0.97	1.25(0.73–2.15)	0.42	1.03(0.83–1.28)	0.79	1.25(0.73–2.13)	0.42	0.92
rs10268496	T/G	All	1200/951	596/510	100/70	0.92(0.79–1.07)	0.28	1.07(0.77–1.49)	0.70	0.94(0.81–1.08)	0.38	1.1(0.79–1.53)	0.57	0.81
		Colon	678/951	342/510	62/70	0.94(0.79–1.12)	0.49	1.18(0.81–1.71)	0.39	0.97(0.82–1.15)	0.72	1.2(0.83–1.74)	0.33	0.79
		Rectum	364/951	175/510	25/70	0.88(0.7–1.09)	0.24	0.86(0.53–1.41)	0.55	0.87(0.71–1.08)	0.21	0.9(0.55–1.46)	0.67	0.37

^a^Numbers may not add up 100% to genotyping failure, covariate missing values or DNA depletion

^b^MM vs Mm = Common homozygous carriers vs heterozygous; MM vs mm = Common homozygous vs rare homozygous; MM vs Mm + mm = Common homozygous vs heterozygous + rare homozygous (Dominant Model); MM + Mm vs mm = Common homozygous + heterozygous vs rare homozygous. Odds Ratio (95% confidence interval).All analysis are adjusted for age, gender and country of origin

### Bioinformatic analysis

We used three bioinformatic tools to identify possible functional properties of rs1525489, the SNP showing the best results from a statistical point of view. RegulomeDB showed a score of six (which means minimal binding evidence). Using HaploReg we did not observe any potentially relevant data. GTEx database did not show any statistically significant eQTLs between the SNP and nearby genes in any of the tissues present in the database.

## Discussion

To further expand our knowledge on CRC genetic susceptibility we investigated whether polymorphic variants within *TAS2R16* could have a role in the etiology of the disease. Our hypothesis finds its rationale on several epidemiologic evidences that polymorphic variants within taste receptor genes could be associated with risk of cancer development. For instance *TAS2R38* SNPs were found to be associated with risk of developing CRC in two different populations of Caucasian origin [23], while SNPs within *TAS2R38* and *TAS1R*s were associated with increased risk of gastric cancer [44, 45]. In this study we examined the genetic variability of *TAS2R16* gene because the receptor binds different compounds including salicin, a natural anti-inflammatory substance which is very similar to aspirin [32, 33]. One of the strongest risk factors for CRC is represented by chronic inflammation especially arisen from diseases that trigger a continuous inflammatory responses such as ulcerative colitis and Crohn’s disease [2]. Aspirin treatment reduces the risk of CRC [2] and therefore genetic variants that modulate the efficiency or the expression of the receptor may play a role in CRC development. Since polymorphisms in taste genes are generally functional as reflected in their effect in a multitude of phenotypes, it is reasonable to hypothesize a possible link between *TAS2R16* allelic variants and CRC risk. To perform our analysis 6 SNPs tagging the common variants present in the region of the gene under examination were chosen. The selection was mainly based on linkage disequilibrium scores between all the variants of the region. Our results seem to suggest no association between the selected polymorphism and risk of developing CRC. The signal closest to the significance threshold of *P* < 0.05 was observed for rs1525489. For this SNP stratifying the analysis for country of origin we found in the Lithuanian and in the Spanish sub-populations a tendency for individuals with at least 1 C allele of the rs1525489 polymorphism to have and increased risk of developing CRC (P 0.047 and *P* = 0.051, respectively). However, these associations were not statistically significant following Bonferroni’s correction. A possible explanation for this association is that this SNP interact with a lifestyle factor or a dietary habit a factor that we do not account for in the analysis. However, this difference maybe just due to statistical fluctuations. Schembre and colleagues have performed a study investigating two *TAS2R16* polymorphic variants (rs846672 and rs846664) in relation to the risk of developing colorectal adenoma [24]. Even though the study is rather small (914 cases of three different ethnic groups) their results are concordant with ours, suggesting an overall no effect of the two variants in the disease. The rs846664 SNP is monomorphic in Caucasian and therefore was not typed in our study while rs846672 is in complete LD with rs860170 that was used in the present study (r^2^ = 1 in European Hapmap Ceu). When analyzing separately colon and rectum, we observed an association between the minor allele of the rs1525489 and an increased risk of developing specifically rectal cancer. This association was showed to have a marginal/borderline statistically significance also after Bonferroni’s correction (*P* = 0.0071). However, this finding must be taken with caution since it is the result of a stratified analysis, on a limited sample size, and it is also difficult to explain from a biological point of view. In fact, the bioinformatics tools did not reveal any possible functional effect for this SNP nor for the variants in LD with it. We acknowledge that the present study has several limitations. We could not collect information on aspirin use and therefore we could not analyze any possible interaction with the SNP analyzed. Moreover, cases and controls are not individually matched for age and gender, however we have adjusted all the analyses for these two variables to minimize their possible effect. We had a power greater than 80% (considering an alpha of 0.05/6 = 0.008) to find an association with an OR of 1.25 or higher for all the SNPs in analysis with the exception of rs1525489 that had a low MAF and for which we had the power to find OR greater than 1.53. An OR of 1.25 is compatible with the ORs found by GWAS in CRC [3]. Using a tagging approach, we have covered most of the common variability of the gene region excluding the possibility of having missed an association with a common but un-typed variant.

## Conclusions

Thus, although we have to be very cautious with the results, our data suggest that polymorphisms of the *TAS2R16* gene do not have a strong influence on colon cancer susceptibility, but that the study should be replicated in large cohorts to better evaluate the effect of rs1525489 on the risk of rectal cancer.

## Additional files

Additional file 1: Table S1. Description of data stratified analysis frequencies, call rate and Hardy-Weinberg equilibrium values of each polymorphism. (DOCX 19 kb)

Additional file 2: Table S2. Description of data Association between colon/rectal cancer risk and SNPs in the *TAS2R16* region considering only the Czech Republic. (DOCX 17 kb)

Additional file 3: Table S3. Description of data Association between colon/rectalcancer risk and SNPs in the *TAS2R16* region considering only Lithuania. (DOCX 18 kb)

Additional file 4: Table S4. Description of data Association between colon/rectalcancer risk and SNPs in the *TAS2R16* region considering only Italy. (DOCX 17 kb)

Additional file 5: Table S5. Description of data Association between colon/rectal cancer risk and SNPs in the *TAS2R16* region considering only Spain. (DOCX 17 kb)

## Abbreviations

CI: Confidence interval
CRC: Colorectal cancer
eQTL: Expression quantitative trait loci
GWAS: Genome-wide association studies
HWE: Hardy-Weinberg equilibrium
LD: Linkage disequilibrium
MAF: Minor allele frequency
OR: Odds ratio
SNP: Single nucleotide polymorphism
