# Supplementary material for: Association between polymorphisms of TAS2R16 and susceptibility to colorectal cancer
Source: BMC Gastroenterol. 2017 Sep 15;17:104. doi: 10.1186/s12876-017-0659-9 (PMC5603047; doi:10.1186/s12876-017-0659-9)
Supplement: Supplementary file 1 — Description of data stratified analysis frequencies, call rate and Hardy-Weinberg equilibrium values of each polymorphism. (DOCX 19 kb) [file 12876_2017_659_MOESM1_ESM.docx]

Supplementary table 1. Frequencies, call rate and Hardy-Weinberg equilibrium values of each polymorphism

| **SNP** | **Alleles (M/m)** | **MAF 1000Genomes** | | | | | **Country** | **Call Rate** | **H-W in Controls** | **MAF in Controls** |
| --- | --- | --- | --- | --- | --- | --- | --- | --- | --- | --- |
|  |  | **EUR** | **CEU** | **FIN** | **IBS** | **TSI** |  |  |  |  |
| rs860170 (Missense) | A/G | 31.40% | 26.30% | 37.90% | 34.60% | 30.80% | Czech Republic | 98.41% | 0.8 | 36.20% |
|  |  |  |  |  |  |  | Lithuania | 99.46% | 0.38 | 36.80% |
|  |  |  |  |  |  |  | Italy | 97.11% | 1 | 31.80% |
|  |  |  |  |  |  |  | Spain | 99.69% | 1 | 30.80% |
|  |  |  |  |  |  |  | Total | 99.03% | 0.73 | 34.60% |
| rs978739 (Upstream) | A/G | 37.20% | 41.90% | 35.90% | 32.20% | 36.90% | Czech Republic | 98.41% | 0.51 | 33.50% |
|  |  |  |  |  |  |  | Lithuania | 99.46% | 0.04 | 29.40% |
|  |  |  |  |  |  |  | Italy | 99.72% | 0.22 | 31.90% |
|  |  |  |  |  |  |  | Spain | 99.69% | 0.66 | 34.70% |
|  |  |  |  |  |  |  | Total | 99.61% | 0.62 | 32.40% |
| rs1357949 (Downstream) | T/C | 28.70% | 30.30% | 25.30% | 28.50% | 29.00% | Czech Republic | 98.94% | 0.72 | 28.20% |
|  |  |  |  |  |  |  | Lithuania | 98.37% | 0.5 | 33.30% |
|  |  |  |  |  |  |  | Italy | 98.85% | 0.81 | 34.20% |
|  |  |  |  |  |  |  | Spain | 99.69% | 0.64 | 31.80% |
|  |  |  |  |  |  |  | Total | 99.35% | 0.72 | 31.20% |
| rs1525489 (Downstream) | T/C | 5.70% | 6.60% | 5.10% | 4.20% | 6.50% | Czech Republic | 97.88% | 1 | 4.60% |
|  |  |  |  |  |  |  | Lithuania | 99.46% | 1 | 2.50% |
|  |  |  |  |  |  |  | Italy | 95.96% | 1 | 3.80% |
|  |  |  |  |  |  |  | Spain | 100.00% | 1 | 1.80% |
|  |  |  |  |  |  |  | Total | 98.16% | 0.3 | 3.10% |
| rs6466849 (Downstream) | G/A | 18.90% | 26.30% | 13.10% | 16.40% | 20.60% | Czech Republic | 98.94% | 0.35 | 19.10% |
|  |  |  |  |  |  |  | Lithuania | 98.91% | 1 | 14.90% |
|  |  |  |  |  |  |  | Italy | 99.72% | 0.04 | 19.40% |
|  |  |  |  |  |  |  | Spain | 100.00% | 0.71 | 18.60% |
|  |  |  |  |  |  |  | Total | 99.68% | 0.84 | 18.20% |
| rs10268496 (Downstream) | G/T | 20.60% | 23.70% | 15.70% | 20.10% | 22.90% | Czech Republic | 100.00% | 0.09 | 21.80% |
|  |  |  |  |  |  |  | Lithuania | 99.46% | 1 | 19.80% |
|  |  |  |  |  |  |  | Italy | 100.00% | 0.22 | 22.70% |
|  |  |  |  |  |  |  | Spain | 99.69% | 0.19 | 21.70% |
|  |  |  |  |  |  |  | Total | 99.87% | 0.84 | 21.20% |
