# Supplementary material for: Association between polymorphisms of TAS2R16 and susceptibility to colorectal cancer
Source: BMC Gastroenterol. 2017 Sep 15;17:104. doi: 10.1186/s12876-017-0659-9 (PMC5603047; doi:10.1186/s12876-017-0659-9)
Supplement: Supplementary file 2 — Description of data Association between colon/rectal cancer risk and SNPs in the TAS2R16 region considering only the Czech Republic. (DOCX 17 kb) [file 12876_2017_659_MOESM2_ESM.docx]

Supplementary table 2. Association between colon/rectal cancer risk and SNPs in the *TAS2R16* region considering only the Czech Republic

| SNP | Alleles (Major/minor) | Site | Case/Control^A^ | | | MM vs Mm^B^ | P value | MM vs mm^B^ | P value | MM vs Mm+mm^B^ | P value | MM+Mm vs mm^B^ | P value | P trend |
| --- | --- | --- | --- | --- | --- | --- | --- | --- | --- | --- | --- | --- | --- | --- |
|  |  |  | MM | Mm | mm |  |  |  |  |  |  |  |  |  |
| rs860170 | A/G | All | 383/277 | 432/312 | 158/98 | 1.06(0.85-1.32) | 0.62 | 1.15(0.84-1.58) | 0.37 | 1.08(0.88-1.33) | 0.47 | 1.12(0.84-1.5) | 0.44 | 0.40 |
|  |  | Colon | 231/277 | 261/312 | 96/98 | 1.03(0.8-1.32) | 0.81 | 1.21(0.85-1.7) | 0.29 | 1.07(0.85-1.36) | 0.56 | 1.19(0.86-1.63) | 0.29 | 0.43 |
|  |  | Rectum | 106/277 | 128/312 | 41/98 | 1.15(0.83-1.58) | 0.40 | 1.12(0.72-1.76) | 0.61 | 1.14(0.84-1.54) | 0.39 | 1.04(0.69-1.58) | 0.84 | 0.62 |
| rs978739 | A/G | All | 426/311 | 371/306 | 83/70 | 0.93(0.75-1.17) | 0.55 | 0.89(0.62-1.29) | 0.54 | 0.93(0.75-1.14) | 0.48 | 0.92(0.65-1.31) | 0.65 | 0.24 |
|  |  | Colon | 264/311 | 209/306 | 47/70 | 0.83(0.65-1.06) | 0.14 | 0.81(0.53-1.23) | 0.32 | 0.82(0.65-1.04) | 0.11 | 0.88(0.59-1.32) | 0.54 | 0.08 |
|  |  | Rectum | 111/311 | 105/306 | 31/70 | 1.06(0.77-1.47) | 0.71 | 1.25(0.76-2.06) | 0.39 | 1.1(0.81-1.49) | 0.55 | 1.21(0.75-1.95) | 0.43 | 0.59 |
| rs1357949 | T/C | All | 516/350 | 375/280 | 85/58 | 0.89(0.72-1.11) | 0.31 | 0.89(0.61-1.3) | 0.54 | 0.89(0.73-1.1) | 0.28 | 0.93(0.65-1.34) | 0.71 | 0.59 |
|  |  | Colon | 298/350 | 235/280 | 54/58 | 0.97(0.76-1.24) | 0.81 | 1.01(0.67-1.53) | 0.95 | 0.98(0.78-1.23) | 0.86 | 1.03(0.69-1.53) | 0.90 | 0.81 |
|  |  | Rectum | 158/350 | 96/280 | 21/58 | 0.74(0.54-1.01) | 0.06 | 0.69(0.39-1.2) | 0.19 | 0.73(0.54-0.98) | 0.04 | 0.78(0.45-1.34) | 0.37 | 0.11 |
| rs1525489 | T/C | All | 893/173 | 76/17 | 0/0 | 0.86(0.49-1.5) | 0.59 |  |  | 0.86(0.49-1.5) | 0.59 |  |  | 0.61 |
|  |  | Colon | 545/173 | 38/17 | 0/0 | 0.7(0.39-1.29) | 0.25 |  |  | 0.7(0.39-1.29) | 0.25 |  |  | 0.26 |
|  |  | Rectum | 245/173 | 28/17 | 0/0 | 1.16(0.61-2.2) | 0.64 |  |  | 1.16(0.61-2.2) | 0.64 |  |  | 0.64 |
| rs6466849 | G/A | All | 668/456 | 269/213 | 31/19 | 0.91(0.73-1.15) | 0.44 | 1.13(0.62-2.05) | 0.70 | 0.93(0.75-1.16) | 0.53 | 1.16(0.64-2.1) | 0.63 | 0.63 |
|  |  | Colon | 416/456 | 153/213 | 19/19 | 0.81(0.63-1.05) | 0.11 | 1.05(0.54-2.06) | 0.88 | 0.83(0.65-1.07) | 0.15 | 1.12(0.58-2.18) | 0.74 | 0.18 |
|  |  | Rectum | 181/456 | 83/213 | 11/19 | 1.07(0.77-1.48) | 0.68 | 1.48(0.67-3.28) | 0.33 | 1.11(0.81-1.51) | 0.53 | 1.45(0.66-3.19) | 0.36 | 0.66 |
| rs10268496 | T/G | All | 650/435 | 286/225 | 51/30 | 0.83(0.66-1.04) | 0.10 | 0.97(0.59-1.58) | 0.90 | 0.85(0.68-1.05) | 0.13 | 1.03(0.63-1.67) | 0.91 | 0.49 |
|  |  | Colon | 386/435 | 177/225 | 32/30 | 0.88(0.69-1.14) | 0.34 | 1.06(0.62-1.8) | 0.84 | 0.91(0.71-1.15) | 0.42 | 1.1(0.65-1.86) | 0.73 | 0.81 |
|  |  | Rectum | 190/435 | 76/225 | 12/30 | 0.73(0.53-1.02) | 0.06 | 0.71(0.35-1.46) | 0.35 | 0.73(0.54-1) | 0.05 | 0.79(0.39-1.6) | 0.51 | 0.19 |

^B^ Numbers may not add up 100% to genotyping failure, covariate missing values or DNA depletion.

^A^ MM vs Mm= Common homozygous carriers vs heterozygous; MM vs mm= Common homozygous vs rare homozygous; MM vs Mm+mm= Common homozygous vs heterozygous + rare homozygous (Dominant Model); MM+Mm vs mm= Common homozygous + heterozygous vs rare homozygous. Odds Ratio (95% confidence interval).All analysis are adjusted for age, gender and country of origin.
