# Supplementary material for: Association between polymorphisms of TAS2R16 and susceptibility to colorectal cancer
Source: BMC Gastroenterol. 2017 Sep 15;17:104. doi: 10.1186/s12876-017-0659-9 (PMC5603047; doi:10.1186/s12876-017-0659-9)
Supplement: Supplementary file 3 — Description of data Association between colon/rectalcancer risk and SNPs in the TAS2R16 region considering only Lithuania. (DOCX 18 kb) [file 12876_2017_659_MOESM3_ESM.docx]

Supplementary table 3. Association between colon/rectal cancer risk and SNPs in the *TAS2R16* region considering only Lithuania

| SNP | Alleles (Major/minor) | Site | Case/Control^A^ | | | MM vs Mm^B^ | P value | MM vs mm^B^ | P value | MM vs Mm+mm^B^ | P value | MM+Mm vs mm^B^ | P value | P trend |
| --- | --- | --- | --- | --- | --- | --- | --- | --- | --- | --- | --- | --- | --- | --- |
|  |  |  | MM | Mm | mm |  |  |  |  |  |  |  |  |  |
| rs860170 | A/G | All | 69/70 | 84/90 | 18/22 | 1.02(0.61-1.69) | 0.95 | 0.9(0.39-2.08) | 0.80 | 1(0.61-1.63) | 0.99 | 0.89(0.4-1.96) | 0.77 | 0.62 |
|  |  | Colon | 21/70 | 21/90 | 5/22 | 0.84(0.39-1.8) | 0.65 | 0.74(0.21-2.63) | 0.64 | 0.82(0.39-1.71) | 0.59 | 0.82(0.25-2.7) | 0.74 | 0.48 |
|  |  | Rectum | 29/70 | 38/90 | 6/22 | 0.96(0.51-1.83) | 0.91 | 0.75(0.23-2.42) | 0.63 | 0.93(0.5-1.73) | 0.81 | 0.77(0.25-2.35) | 0.64 | 0.57 |
| rs978739 | A/G | All | 85/85 | 79/87 | 14/10 | 0.87(0.53-1.41) | 0.56 | 0.97(0.36-2.63) | 0.96 | 0.88(0.55-1.41) | 0.59 | 1.04(0.4-2.74) | 0.93 | 0.84 |
|  |  | Colon | 22/85 | 22/87 | 4/10 | 0.92(0.43-1.95) | 0.83 | 1.13(0.26-4.92) | 0.88 | 0.94(0.46-1.95) | 0.88 | 1.17(0.28-4.88) | 0.83 | 0.70 |
|  |  | Rectum | 41/85 | 30/87 | 7/10 | 0.74(0.4-1.37) | 0.34 | 1.3(0.41-4.15) | 0.65 | 0.8(0.44-1.45) | 0.47 | 1.5(0.49-4.61) | 0.48 | 0.77 |
| rs1357949 | T/C | All | 75/78 | 86/84 | 16/18 | 1.13(0.68-1.87) | 0.64 | 0.94(0.4-2.18) | 0.88 | 1.09(0.67-1.77) | 0.72 | 0.88(0.39-1.95) | 0.75 | 1.00 |
|  |  | Colon | 18/78 | 26/84 | 4/18 | 1.3(0.6-2.81) | 0.50 | 1.11(0.28-4.35) | 0.89 | 1.27(0.6-2.69) | 0.53 | 0.95(0.26-3.47) | 0.94 | 0.69 |
|  |  | Rectum | 31/78 | 38/84 | 9/18 | 1.1(0.58-2.09) | 0.77 | 1.15(0.42-3.17) | 0.79 | 1.11(0.6-2.05) | 0.74 | 1.09(0.42-2.8) | 0.86 | 0.56 |
| rs1525489 | T/C | All | 158/173 | 17/9 | 1/0 | 1.56(0.6-4.07) | 0.37 |  |  | 1.6(0.62-4.14) | 0.34 |  |  | 0.05 |
|  |  | Colon | 43/173 | 5/9 | 0/0 | 1.6(0.42-6.03) | 0.49 |  |  | 1.6(0.42-6.03) | 0.49 |  |  | 0.16 |
|  |  | Rectum | 68/173 | 9/9 | 1/0 | 2(0.64-6.21) | 0.23 |  |  | 2.09(0.69-6.38) | 0.19 |  |  | 0.02 |
| rs6466849 | G/A | All | 131/131 | 42/46 | 4/4 | 1.03(0.59-1.79) | 0.91 | 0.77(0.15-4.01) | 0.76 | 1.01(0.59-1.72) | 0.98 | 0.77(0.15-3.96) | 0.75 | 0.76 |
|  |  | Colon | 36/131 | 10/46 | 2/4 | 0.86(0.35-2.13) | 0.75 | 1.39(0.17-11.46) | 0.76 | 0.91(0.39-2.15) | 0.84 | 1.43(0.18-11.72) | 0.74 | 0.94 |
|  |  | Rectum | 60/131 | 16/46 | 1/4 | 0.91(0.45-1.85) | 0.79 | 0.5(0.05-5.27) | 0.56 | 0.87(0.43-1.74) | 0.69 | 0.51(0.05-5.36) | 0.57 | 0.33 |
| rs10268496 | T/G | All | 106/117 | 64/58 | 8/7 | 1.3(0.78-2.16) | 0.31 | 1.43(0.46-4.4) | 0.54 | 1.31(0.81-2.14) | 0.27 | 1.3(0.43-3.95) | 0.64 | 0.37 |
|  |  | Colon | 26/117 | 21/58 | 1/7 | 1.54(0.73-3.25) | 0.26 | 1.28(0.14-11.41) | 0.82 | 1.52(0.73-3.16) | 0.26 | 1.09(0.13-9.49) | 0.94 | 0.36 |
|  |  | Rectum | 43/117 | 30/58 | 5/7 | 1.49(0.79-2.8) | 0.21 | 2.1(0.58-7.64) | 0.26 | 1.56(0.85-2.86) | 0.15 | 1.8(0.51-6.39) | 0.36 | 0.14 |

^B^ Numbers may not add up 100% to genotyping failure, covariate missing values or DNA depletion.^A^ MM vs Mm= Common homozygous carriers vs heterozygous; MM vs mm= Common homozygous vs rare homozygous; MM vs Mm+mm= Common homozygous vs heterozygous + rare homozygous (Dominant Model); MM+Mm vs mm= Common homozygous + heterozygous vs rare homozygous. Odds Ratio (95% confidence interval).All analysis are adjusted for age, gender and country of origin.
