# Supplementary material for: Association between polymorphisms of TAS2R16 and susceptibility to colorectal cancer
Source: BMC Gastroenterol. 2017 Sep 15;17:104. doi: 10.1186/s12876-017-0659-9 (PMC5603047; doi:10.1186/s12876-017-0659-9)
Supplement: Supplementary file 4 — Description of data Association between colon/rectalcancer risk and SNPs in the TAS2R16 region considering only Italy. (DOCX 17 kb) [file 12876_2017_659_MOESM4_ESM.docx]

Supplementary table 4. Association between colon/rectal cancer risk and SNPs in the *TAS2R16* region considering only Italy

| SNP | Alleles (Major/minor) | Site | Case/Control^A^ | | | MM vs Mm^B^ | P value | MM vs mm^B^ | P value | MM vs Mm+mm^B^ | P value | MM+Mm vs mm^B^ | P value | P trend |
| --- | --- | --- | --- | --- | --- | --- | --- | --- | --- | --- | --- | --- | --- | --- |
|  |  |  | MM | Mm | mm |  |  |  |  |  |  |  |  |  |
| rs860170 | A/G | All | 152/156 | 132/146 | 35/34 | 0.96(0.67-1.39) | 0.85 | 1.06(0.58-1.91) | 0.86 | 0.98(0.69-1.39) | 0.92 | 1.07(0.61-1.9) | 0.81 | 0.94 |
|  |  | Colon | 89/156 | 77/146 | 18/34 | 0.85(0.55-1.32) | 0.48 | 0.85(0.42-1.74) | 0.66 | 0.85(0.57-1.29) | 0.45 | 0.92(0.46-1.82) | 0.81 | 0.71 |
|  |  | Rectum | 26/156 | 25/146 | 10/34 | 0.95(0.5-1.8) | 0.88 | 1.59(0.65-3.91) | 0.31 | 1.07(0.59-1.94) | 0.82 | 1.63(0.7-3.79) | 0.26 | 0.36 |
| rs978739 | A/G | All | 142/165 | 142/140 | 35/40 | 1.14(0.79-1.65) | 0.48 | 1.12(0.63-1.99) | 0.70 | 1.14(0.8-1.61) | 0.47 | 1.05(0.61-1.82) | 0.86 | 0.61 |
|  |  | Colon | 86/165 | 78/140 | 19/40 | 1.09(0.71-1.68) | 0.69 | 1.1(0.55-2.19) | 0.79 | 1.09(0.73-1.64) | 0.67 | 1.05(0.55-2.04) | 0.88 | 0.95 |
|  |  | Rectum | 30/165 | 22/140 | 10/40 | 0.95(0.5-1.79) | 0.87 | 1.5(0.62-3.62) | 0.37 | 1.07(0.59-1.91) | 0.83 | 1.53(0.66-3.55) | 0.32 | 0.68 |
| rs1357949 | T/C | All | 136/147 | 130/156 | 41/39 | 0.76(0.53-1.11) | 0.16 | 1.02(0.58-1.79) | 0.94 | 0.82(0.57-1.16) | 0.26 | 1.17(0.69-1.98) | 0.56 | 0.91 |
|  |  | Colon | 75/147 | 71/156 | 31/39 | 0.76(0.48-1.19) | 0.23 | 1.39(0.75-2.58) | 0.30 | 0.88(0.58-1.34) | 0.56 | 1.6(0.9-2.85) | 0.11 | 0.29 |
|  |  | Rectum | 30/147 | 22/156 | 4/39 | 0.62(0.33-1.19) | 0.15 | 0.53(0.16-1.7) | 0.28 | 0.61(0.33-1.13) | 0.11 | 0.67(0.22-2.06) | 0.48 | 0.12 |
| rs1525489 | T/C | All | 290/307 | 24/25 | 0/0 | 0.95(0.48-1.87) | 0.87 |  |  | 0.95(0.48-1.87) | 0.87 |  |  | 0.96 |
|  |  | Colon | 165/307 | 15/25 | 0/0 | 1.06(0.48-2.31) | 0.89 |  |  | 1.06(0.48-2.31) | 0.89 |  |  | 0.75 |
|  |  | Rectum | 55/307 | 7/25 | 0/0 | 1.75(0.65-4.7) | 0.27 |  |  | 1.75(0.65-4.7) | 0.27 |  |  | 0.32 |
| rs6466849 | G/A | All | 192/230 | 98/96 | 12/19 | 1.34(0.91-1.98) | 0.14 | 1.01(0.44-2.31) | 0.98 | 1.29(0.89-1.87) | 0.18 | 0.92(0.41-2.08) | 0.85 | 0.73 |
|  |  | Colon | 119/230 | 46/96 | 9/19 | 1.06(0.66-1.71) | 0.80 | 1.13(0.45-2.83) | 0.79 | 1.07(0.69-1.68) | 0.75 | 1.11(0.45-2.75) | 0.82 | 0.71 |
|  |  | Rectum | 35/230 | 20/96 | 1/19 | 1.6(0.83-3.1) | 0.16 | 0.33(0.04-2.85) | 0.32 | 1.37(0.72-2.61) | 0.33 | 0.29(0.03-2.41) | 0.25 | 0.96 |
| rs10268496 | T/G | All | 184/211 | 112/113 | 21/22 | 1.1(0.76-1.59) | 0.61 | 1.05(0.52-2.14) | 0.89 | 1.09(0.77-1.55) | 0.62 | 1.01(0.51-2.04) | 0.97 | 0.50 |
|  |  | Colon | 104/211 | 61/113 | 17/22 | 1.07(0.69-1.66) | 0.78 | 1.43(0.66-3.09) | 0.36 | 1.13(0.75-1.7) | 0.57 | 1.4(0.66-2.97) | 0.38 | 0.24 |
|  |  | Rectum | 40/211 | 22/113 | 0/22 | 1.04(0.57-1.92) | 0.90 |  |  | 0.87(0.48-1.59) | 0.66 |  |  | 0.23 |

^B^ Numbers may not add up 100% to genotyping failure, covariate missing values or DNA depletion.

^A^ MM vs Mm= Common homozygous carriers vs heterozygous; MM vs mm= Common homozygous vs rare homozygous; MM vs Mm+mm= Common homozygous vs heterozygous + rare homozygous (Dominant Model); MM+Mm vs mm= Common homozygous + heterozygous vs rare homozygous. Odds Ratio (95% confidence interval).All analysis are adjusted for age, gender and country of origin.
