# Supplementary material for: Association between polymorphisms of TAS2R16 and susceptibility to colorectal cancer
Source: BMC Gastroenterol. 2017 Sep 15;17:104. doi: 10.1186/s12876-017-0659-9 (PMC5603047; doi:10.1186/s12876-017-0659-9)
Supplement: Supplementary file 5 — Description of data Association between colon/rectal cancer risk and SNPs in the TAS2R16 region considering only Spain. (DOCX 17 kb) [file 12876_2017_659_MOESM5_ESM.docx]

Supplementary table 5. Association between colon/rectal cancer risk and SNPs in the *TAS2R16* region considering only Spain

| SNP | Alleles (Major/minor) | Site | Case/Control^A^ | | | MM vs Mm^B^ | P value | MM vs mm^B^ | P value | MM vs Mm+mm^B^ | P value | MM+Mm vs mm^B^ | P value | P trend |
| --- | --- | --- | --- | --- | --- | --- | --- | --- | --- | --- | --- | --- | --- | --- |
|  |  |  | MM | Mm | mm |  |  |  |  |  |  |  |  |  |
| rs860170 | A/G | All | 181/150 | 198/133 | 34/30 | 1.23(0.9-1.67) | 0.20 | 0.91(0.53-1.56) | 0.74 | 1.17(0.87-1.57) | 0.31 | 0.82(0.49-1.38) | 0.46 | 0.57 |
|  |  | Colon | 106/150 | 128/133 | 22/30 | 1.37(0.96-1.94) | 0.08 | 1.01(0.55-1.85) | 0.98 | 1.3(0.93-1.82) | 0.13 | 0.86(0.48-1.54) | 0.61 | 0.31 |
|  |  | Rectum | 70/150 | 65/133 | 11/30 | 1.03(0.68-1.56) | 0.89 | 0.76(0.36-1.6) | 0.47 | 0.98(0.66-1.45) | 0.92 | 0.75(0.36-1.54) | 0.43 | 0.75 |
| rs978739 | A/G | All | 177/132 | 185/145 | 50/36 | 0.97(0.7-1.32) | 0.83 | 1.05(0.65-1.71) | 0.83 | 0.98(0.73-1.33) | 0.91 | 1.07(0.68-1.7) | 0.76 | 0.98 |
|  |  | Colon | 115/132 | 113/145 | 27/36 | 0.92(0.64-1.3) | 0.62 | 0.87(0.5-1.52) | 0.63 | 0.91(0.65-1.27) | 0.57 | 0.91(0.53-1.55) | 0.73 | 0.49 |
|  |  | Rectum | 58/132 | 65/145 | 23/36 | 1.02(0.67-1.57) | 0.92 | 1.47(0.8-2.71) | 0.22 | 1.11(0.74-1.66) | 0.61 | 1.45(0.82-2.57) | 0.20 | 0.33 |
| rs1357949 | T/C | All | 200/144 | 170/139 | 38/30 | 0.88(0.65-1.21) | 0.44 | 0.9(0.53-1.53) | 0.70 | 0.89(0.66-1.19) | 0.43 | 0.96(0.58-1.59) | 0.86 | 0.50 |
|  |  | Colon | 121/144 | 111/139 | 21/30 | 0.96(0.68-1.37) | 0.84 | 0.81(0.44-1.5) | 0.51 | 0.94(0.67-1.31) | 0.70 | 0.83(0.46-1.49) | 0.53 | 0.57 |
|  |  | Rectum | 73/144 | 55/139 | 16/30 | 0.79(0.52-1.2) | 0.27 | 1.08(0.55-2.11) | 0.83 | 0.84(0.56-1.25) | 0.38 | 1.2(0.63-2.29) | 0.58 | 0.63 |
| rs1525489 | T/C | All | 387/303 | 28/11 | 0/0 | 2.04(1-4.19) | 0.05 |  |  | 2.04(1-4.19) | 0.05 |  |  | 0.05 |
|  |  | Colon | 239/303 | 19/11 | 0/0 | 2.3(1.07-4.95) | 0.03 |  |  | 2.3(1.07-4.95) | 0.03 |  |  | 0.04 |
|  |  | Rectum | 137/303 | 9/11 | 0/0 | 1.93(0.78-4.8) | 0.16 |  |  | 1.93(0.78-4.8) | 0.16 |  |  | 0.19 |
| rs6466849 | G/A | All | 285/207 | 115/97 | 15/10 | 0.87(0.63-1.2) | 0.40 | 1.06(0.46-2.4) | 0.90 | 0.89(0.65-1.21) | 0.46 | 1.1(0.49-2.49) | 0.82 | 0.57 |
|  |  | Colon | 177/207 | 75/97 | 6/10 | 0.9(0.63-1.3) | 0.58 | 0.65(0.23-1.82) | 0.41 | 0.88(0.62-1.25) | 0.47 | 0.67(0.24-1.87) | 0.44 | 0.43 |
|  |  | Rectum | 100/207 | 37/97 | 9/10 | 0.79(0.51-1.25) | 0.32 | 1.85(0.73-4.73) | 0.20 | 0.89(0.59-1.36) | 0.61 | 1.98(0.78-5.01) | 0.15 | 0.94 |
| rs10268496 | T/G | All | 260/188 | 134/114 | 20/11 | 0.84(0.61-1.15) | 0.27 | 1.28(0.6-2.74) | 0.53 | 0.88(0.65-1.19) | 0.40 | 1.36(0.64-2.89) | 0.42 | 0.74 |
|  |  | Colon | 162/188 | 83/114 | 12/11 | 0.83(0.58-1.19) | 0.31 | 1.2(0.51-2.81) | 0.67 | 0.87(0.61-1.22) | 0.41 | 1.28(0.55-2.97) | 0.56 | 0.71 |
|  |  | Rectum | 91/188 | 47/114 | 8/11 | 0.85(0.56-1.3) | 0.45 | 1.49(0.58-3.84) | 0.41 | 0.91(0.6-1.36) | 0.64 | 1.58(0.62-4.02) | 0.34 | 0.96 |

^B^ Numbers may not add up 100% to genotyping failure, covariate missing values or DNA depletion.

^A^ MM vs Mm= Common homozygous carriers vs heterozygous; MM vs mm= Common homozygous vs rare homozygous; MM vs Mm+mm= Common homozygous vs heterozygous + rare homozygous (Dominant Model); MM+Mm vs mm= Common homozygous + heterozygous vs rare homozygous. Odds Ratio (95% confidence interval).All analysis are adjusted for age, gender and country of origin.
